# Estimation of Pubertal Growth Spurt Parameters in Children and Adolescents Living at Moderate Altitude in Colombia

**DOI:** 10.3389/fendo.2021.718292

**Published:** 2021-09-08

**Authors:** Marco Antonio Cossio-Bolaños, Ruben Vidal-Espinoza, Juan Minango-Negrete, Pedro R. Olivares, Luis Urzua-Alul, Luis Felipe Castelli Correia de Campos, Jose Fuentes-López, Lucila Sanchez-Macedo, Edilberto Diaz-Bonilla, Claudia Torres-Galvis, Rossana Gomez-Campos

**Affiliations:** ^1^Department of Physical Activity Sciences, Faculty of Education Sciences, Catholic University of the Maule, Talca, Chile; ^2^Universidad Católica Silva Henríquez, Santiago, Chile; ^3^Instituto Superior Universitario Rumiñahui, Sangolqui, Quito, Ecuador; ^4^Faculty of Education, Psychology and Sport Sciences University of Huelva, Huelva, Spain; ^5^EFISAL Research Group, Universidad Autonoma de Chile, Talca, Chile; ^6^Escuela de Kinesiología, Facultad de Salud, Universidad Santo Tomás, Santiago, Chile; ^7^Department of Education Sciences, Faculty of Education and Humanities, University of Bío Bío, Chillan, Chile; ^8^Instituto de Investigación en Ciencias de la Educación (IICE), Universidad Nacional del Altiplano de Puno, Puno, Perú; ^9^Universidad Pedagógica y Tecnológica de Colombia, Tunja, Colombia; ^10^Department of Educational Diversity and Inclusivity, Catholic University of the Maule, Talca, Chile

**Keywords:** growth velocity, stature, final height, Preece–Baines function, children

## Abstract

**Objective:**

Knowledge of the biological parameters of pubertal growth spurt allows verification of secular changes and exploration of the timing of puberty. The aim of the study was to estimate final height, age at peak height velocity (APHV), and peak height velocity PHV (cm/y) in children and adolescents living at moderate altitude in Colombia.

**Methods:**

A cross-sectional study was designed in 2.295 schoolchildren from Bogotá (Colombia) with an age range from 5.0 to 18.9 years. Height (cm) was assessed. Preece–Baines model 1 (1PB) was used to make inferences about mathematical and biological parameters.

**Results:**

The five mathematical parameters estimated in general have reflected quality in the fit to the model, reflecting a small residual error. Final height was reached in boys at 170.8 ± 0.4 cm and in girls at 157.9 ± 0.2 cm. APHV was estimated at 12.71 ± 0.1 years in boys and 10.4 ± 0.2 years in girls. Girls reached APHV 2.2 years earlier than boys. In relation to PHV (cm/y), boys reached higher growth speed in height (7.4 ± 0.4 cm/y), and in girls it was (7.0 ± 0.2 cm/y).

**Conclusion:**

It was determined that final height was reached at 170.8 ± 0.4 cm in boys and 157.9 ± 0.2 cm in girls, and APHV (years) and PHV (cm/ye) were reached relatively early and with average peak velocity similar to Asian and Western populations. A large-scale longitudinal study is needed to confirm these findings.

## Introduction

Human growth is traditionally conceived as a goal-seeking process regulated by genes, nutrition, health, and the state of an individual’s social and economic environment (1, 2). It is characterized by extraordinary plasticity and population heterogeneity (3).

It is considered fundamental for planning the promotion of health programs and serves as a basis for pediatricians to assess diseases with possible growth disturbances (4). Its assessment can be made from longitudinal, cross-sectional, mixed longitudinal, or linked longitudinal studies (1). However, to determine growth velocity, a longitudinal study should be used before a cross-sectional study (5).

In fact, conducting a longitudinal study involves long periods of time, often years or decades (6), which makes the process of collecting data continuously difficult although, in general, in recent years, the estimation of peak growth velocity and pubertal growth spurt parameters in children and adolescents from various parts of the world (4, 7, 8) have been determined by mathematical models, specifically Preece–Baines (9) model 1 (1PB).

Consequently, based on the fact that children and adolescents have varied growth rates (1) in general, girls average a maximum height velocity of 9 cm/y at age 12 years, and boys, on average, reach a maximum height velocity of 10.3 cm/y at age 14 years (1, 10, 11) this study assumes that children and adolescents living at moderate altitude in Bogotá, Colombia, could reflect similar growth patterns to those reported by the literature.

Therefore, the objective of the study was to estimate final height, age at peak height velocity (APHV), and peak height velocity (PHV) (cm/y) in children and adolescents living at moderate altitude in Colombia. These parameters are determined by means of 1PB (9) as an alternative means of approximating longitudinal growth patterns.

## Materials and Methods

### Type of Study and Sample

A descriptive (cross-sectional) study was carried out in 2295 schoolchildren (1175 boys and 1120 girls) in Bogotá (Colombia). This city is the capital of Colombia and is located 2640 m above sea level. The sample selection was nonprobabilistic (quotas). The age range was 5 to 18.9 years.

The schoolchildren participated voluntarily in the study. Parents signed a consent form, and children and adolescents signed an informed consent form. Schoolchildren who were within the school age range (primary and secondary level) and those who completed the anthropometric evaluations were included. Those with any type of physical injury and children who were foreigners were excluded. The study was conducted in accordance with Resolution 8430 of the Colombian Ministry of Health and Social Protection as well as with the Declaration of Helsinki for human beings. The Human Development Index according to the United Nations Development Program (12) highlighted that, for 2012, in Colombia was 0.719 and, according to Global Data Lab (13), for Bogotá was 0.773.

### Techniques and Procedures

Height was measured at the school facilities during school hours (Monday to Friday) during the month of October 2013. Date of birth information (day, month, and year) was requested from the school administration. The date of the evaluation was recorded, and with both records, the decimal age was obtained.

The height evaluation was carried out according to the suggestions described by Ross and Marfell-Jones (14). The procedure consisted of measuring height (cm) without shoes and with as little clothing as possible (only a light T-shirt and shorts). A portable stadiometer (Seca Gmbh & Co. KG, Hamburg, Germany) with an accuracy of 0.1 cm, according to the Frankfort plane, was used. To guarantee the technical error of stature measurement, it was measured twice, reflecting intra- and inter-observer values from *R* = .85 to.92.

The *Z*-score was calculated for each age and sex according to the standards described by the World Health Organization (15). The 1PB (9) nonlinear regression model 1 was used to infer the mathematical and biological parameters of height growth velocity and growth spurt for both sexes.

### Statistics

Normality of data was verified by the Kolmogorov–Smirnov (K-S) test. After conforming to normality, descriptive statistics (mean, standard deviation, range) were calculated. Differences between both sexes were verified by means of the *t-*test for independent samples. To estimate the mathematical and biological parameters, the 1PB model was used. Five parameters were calculated according to the following equation:


$$h=h_{1}-\frac{2(h_{1}-h_{\theta })}{e^{s_{0}(t-\theta )}+e^{s_{1}(t-\theta )}}$$

Here, h1: final height (cm), hθ and θ: average height (cm) and age (years) for height on the decreasing slope of the PHV, s0 and s1: prepubertal and pubertal rate constants controlling growth rate (cm/y). The calculations and graphs of the curves were obtained by means of the computer program implemented in the software R.

## Results

**Figure 1:**
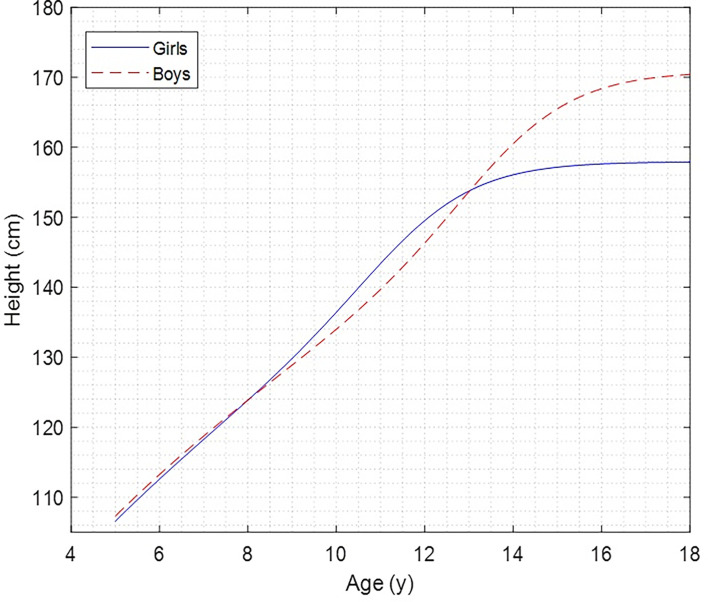
Height growth curve of children and adolescents by age and sex.

The X ± SD values of heights of children and adolescents aligned by age and sex are shown in **Table 1**. There were no significant differences between both sexes from 5 to 8 years old and at 12 and 13 years old although, from 9 to 11 years old and 14 to 18 years old, the differences are significant (*p* **<**.05). The *Z* **-**score values in boys ranged from 0.20 to -0.82 cm and in girls from 0.32 to -0.78 cm. The height growth curve from 5 to 18 years of age for both sexes is observed in **Figure 1**. A similar growth pattern is observed until 8 years of age; then, from 9 to 11 years of age, girls showed greater height than boys (*p* **<**.05), and from 14 to 18 years of age, boys evidenced greater height than girls (*p* **<**.05).

**Table 1:** Descriptive values (mean ± SD) and Z-score of the height of children and adolescents in Colombia.

Age (y)		Boys		Z score			Girls		Z score		p (height)
	n	X	SD	X	SD	n	X	SD	X	SD	
5	48	111.1	4.77	0.20	1.05	48	110.82	4.55	0.28	0.95	.811
6	78	115.5	4.61	-0.07	0.94	63	115.18	5.81	0.03	1.14	.695
7	91	120.9	5.59	-0.16	1.06	88	121.51	4.59	0.14	0.85	.366
8	92	126.6	5.49	-0.12	0.96	82	127.48	6.43	0.19	1.12	.248
9	120	131.41	5.61	-0.18	0.94	90	133.19	6.39	0.16	1.04	.019
10	85	136.3	6.6	-0.20	1.03	78	140.49	6.86	0.32	1.08	.000
11	107	143.6	7.57	0.11	1.13	100	146.51	6.55	0.23	0.99	.007
12	99	149.4	7.22	0.06	1.02	90	151.32	6.46	0.00	0.92	.085
13	86	157.5	8.41	0.19	1.13	91	155.52	4.81	-0.10	0.71	.089
14	98	164.4	6.85	0.18	0.90	96	156.90	5.64	-0.41	0.82	.000
15	90	167.0	6.41	-0.25	0.82	114	158.07	5.76	-0.51	0.84	.000
16	99	169.0	5.83	-0.50	0.75	108	157.11	5.17	-0.78	0.77	.000
17	63	171.0	5.14	-0.52	0.67	53	157.59	5.18	-0.77	0.77	.000
18	19	169.9	8.4	-0.82	1.12	19	157.82	6.13	-0.77	0.91	.000

X, Mean; SD, Standard deviation; P, Probability (p <.05).

The mathematical and biological parameters of the height of Colombian children and adolescents are shown in **Table 2**. The five mathematical parameters estimated by the 1PB model in general have reflected quality in the fit to the model, being the residual small (residual SE) in both sexes (0.241 in male and 0.203 in female). Final height (hi) (170.849 ± 0.434 cm) and peak height velocity size (hθ) (156.817 ± 0.495 cm) of boys were significantly higher relative to girls (hi = 157.934 ± 0.211 cm) and (hθ = 145.691 ± 0.863 cm) (*p* <.05).

**Table 2:** Mathematical and biological parameters of stature, estimated by 1Preece-Baines (1PB) model.

PB model parameters	Boys		Girls	
	X	SE	X	SE
Mathematical parameters				
s0	0.10	0.01	0.12	0.01
s1	0.90	0.06	0.93	0.06
θ	13.44	0.10	11.35	0.16
hθ	156.82	0.50	145.69*	0.86
h1	170.85	0.43	157.93*	0.21
Residual SE	0,24		0,20	
Biological Parameters				
Age at peak velocity (y)	12.71	0.13	10.42*	0.20
Peak height velocity (cm/y)	7.43	0.42	7.01	0.19

X, mean; SE, Standard error; h1, final height (cm); hθ and θ, average height (cm); and age (years) for height on the decreasing slope of the PHV, s0 and s1, prepubertal and pubertal rate constants controlling growth rate (cm/y). *Significant difference in relation to girls (p <.05).

**Figure 2:**
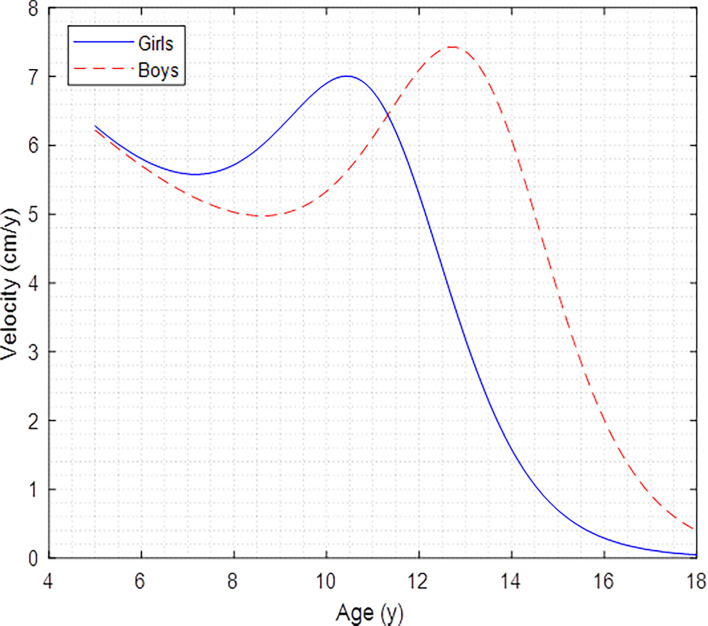
Peak height velocity curves of Colombian children and adolescents of both sexes.

In boys, the APHV was estimated at 12.708 ± 0.131 years, and in girls it was at 10.422 ± 0.195 years. In both ages, there were significant differences (*p* **<**.05). Girls reached APHV 2.23 years earlier than boys. In relation to PHV (cm/y), boys reached higher growth velocity in height, which was 7.430 ± 0.419 cm/y; however, in girls, it was 7.006 ± 0.186 cm/y. These values of APHV and PHV can be seen in **Figure 2**.

## Discussion

The results of the study evidence that schoolchildren living at moderate altitude in Bogotá (Colombia) reflect a final height in boys of 170.84 ± 0.43 cm and in girls of 157.93 ± 0.21 cm. These values are relatively similar to those reported by a recent study conducted in Colombia by Durán et al. (16) from birth to 20 years of age (in which, for boys, it was 172.3 cm and 159.4 cm for girls), covering cities such as Bogotá, Medellín, Cali, and Barranquilla that represent 92% of the Colombian population. In fact, Meisel-Roca et al. (17) highlight that disparities in average height between departments in Colombia were reduced in recent years, and the gap between regions has been closing throughout the century, especially due to the positive evolution of height during the period 1965–1990 when several ethnic groups in Colombia increased their height positively (18).

In relation to international studies, the final heights observed in our study are relatively similar to those reported in a cross-sectional study in Argentine children (19) and in British children through a longitudinal study (20) although Peruvian children and adolescents (8) reflect lower heights than those reported in this study (164.24 ± 0.67 cm in boys and 153.06 ± 0.29 cm in girls).

Regarding APHV, this study reports that children in Bogotá reflect 12.71 ± 0.13 y in boys and 10.42 ± 0.19 y in girls; i.e., these values presented prematurely (~1.6 years) earlier than indicated by the literature, in which the peak is reached around 14 years in boys and 12 years in girls approximately (11, 21).

The values observed in this study show earlier ages for APHV with respect to the study conducted in Yopal (Colombia), reporting 11.1 y in girls and 13.6 y in boys (22). On the other hand, when compared with international research, the results of this study are similar to those described in Chinese (4), Peruvian (8), and Taiwanese (23) children, in studies that used the 1BP model to estimate APHV in cross-sectional samples. Indeed, in all three studies, girls evidenced APHVs 2 years earlier than boys as observed in this study and the literature, respectively (11, 21).

In general, the estimation of APHVs cross-sectionally in the children in our study and in the Chinese, Peruvians, and Taiwanese children occurred prematurely, suggesting that values obtained in cross-sectional samples may underestimate APHVs. In fact, these divergences generally occur in cross-sectional samples, especially at puberty, when the shape of the underlying individual mean “growth” curve defines temporal changes in the timing of the cross-sectional distribution, whose data distribution may be transiently skewed (24).

On the other hand, PHV was higher in boys from Bogotá (7.43 ± 0.42 y) than girls from the same region (7.00 ± 0.19 y). These velocity patterns (cm/y) were more accelerated than similar ones from Yopal (Colombia) evidencing 6.96 cm/y in boys and 6.57 cm/y in girls (22). However, when compared with international research, some studies report values similar to our findings, such as, for example, those evidenced by Chinese (4) and Japanese (25) children, although some others have described height velocity higher than 7.5 cm/y in girls and 8.2 cm/y in Guatemalan and British children (20, 26), respectively.

Consequently, the cross-sectional studies using the 1PB model described above and the results obtained in this study report much lower velocities than those described in longitudinal studies (5, 11). These results are possibly due to some complications that cross-sectional studies usually present. For example, it is necessary to estimate derivatives (velocity and acceleration) from height measurements (27), which possibly the 1PB model could incur in a lower estimation of the velocity at peak stretch in relation to the models used in longitudinal studies (19, 20). However, it is also possible that cross-sectional studies show different cohorts in each of their ages, which could be exposed to different environmental conditions, which could force the model to adjust the data, consequently producing biases in the results (20).

In essence, in this study, we verified that final statures among adolescents differ with other study samples, including APHV and PHV varying between regions, so these findings confirm variations in physical growth potential between populations, which is determined by intergenerational factors—not only by genes transmitted to generations, but also to environmental living conditions (18).

The results of this study are relevant because, during the last century, the evolution of the biological standard of living in Colombia was a success story from the point of view of the secular increase in height and the reduction of socioeconomic inequality (28), including the increase in the human development index in recent years (13), all of which could be factors that explain in part the PHV reached prematurely and the reduction of disparities in final height compared to other populations.

Overall, despite the reduction of physical growth differences in Latin American regions and the improvement of living conditions, it remains to date a major public health problem as 5.1 million stunted affected children have been evidenced up to 2017 (29). In addition, data on physical growth and pubertal growth spurt parameters of children and adolescents in these regions remain scarce, so future studies should focus their research on three ethnic groups, namely indigenous, Afro-descendants, and Europeans with considerable degrees of miscegenation (30) as well as including Colombian children and adolescents living at sea level, at moderate altitude, and in tropical regions. This information may be useful to professionals and researchers working in auxology in general.

The study presents some strengths, for example, to our knowledge, this is the first cross-sectional study that was conducted in Bogotá (Colombia) on a large scale, estimating final height, APHVs, and PHVs in children and adolescents, as the results obtained provide relevant information to professionals working in clinical and epidemiological contexts. In addition, the biological parameters obtained can serve as a reference to compare with other regions, verify secular changes, and explore the timing of puberty among boys and girls, respectively.

Nevertheless, it was not possible to evaluate socioeconomic variables of the sample studied; however, it is assumed that children studying in Colombian state schools generally belong to a middle socioeconomic condition. Also, the selection of the nonprobabilistic sample does not allow generalizing the data to other contexts, and the 1PB model could underestimate the biological parameters, especially if cross-sectional data (20) such as in the present study are analyzed. Therefore, it is recommended that future studies should take these aspects into account and should be analyzed with caution.

In conclusion, a cross-sectional study was conducted on a large sample size of children living in Bogotá (Colombia) to determine growth patterns adjusted by the 1PB model. It was determined that final height size was reached at 170.84 ± 0.43 cm in boys and 157.93 ± 0.21 cm in girls, and APHV and PHV (cm/y) were reached relatively early in life and at average peak velocities similar to Asian and Western populations. A large-scale longitudinal study is needed to confirm these findings.

## Data Availability Statement

The raw data supporting the conclusions of this article will be made available by the authors, without undue reservation.

## Ethics Statement

The studies involving human participants were reviewed and approved by Technological and Pedagogical University of Colombia. Written informed consent to participate in this study was provided by the participants’ legal guardian/next of kin.

## Author Contributions

RG-C, MC-B, and RV-E conceived and designed the experiments. ED-B and CT-G performed the experiments. JM-N, MC-B, RG-C, PO, LU-A, and FC-C analyzed the data. MC-B, RG-C, RV-E, JF-L, and LS-M wrote, reviewed, and edited the paper. All authors contributed to the article and approved the submitted version.

## Conflict of Interest

The authors declare that the research was conducted in the absence of any commercial or financial relationships that could be construed as a potential conflict of interest.

## Publisher’s Note

All claims expressed in this article are solely those of the authors and do not necessarily represent those of their affiliated organizations, or those of the publisher, the editors and the reviewers. Any product that may be evaluated in this article, or claim that may be made by its manufacturer, is not guaranteed or endorsed by the publisher.
